# Impact of the Food and Drug Administration enforcement policy on flavored e-cigarettes on the online popularity of disposable e-cigarettes: analyses of Google search query data

**DOI:** 10.1186/s12889-022-14367-3

**Published:** 2022-10-19

**Authors:** Ellen Boakye, Omar Dzaye, John Erhabor, Ngozi Osuji, Olufunmilayo Obisesan, Albert D. Osei, Aruni Bhatnagar, Rose Marie Robertson, Michael J. Blaha

**Affiliations:** 1 Johns Hopkins Ciccarone Center for Prevention of Cardiovascular Disease, 600 N Wolfe St, Blalock 524, Baltimore, MD 21287 USA; 2 The American Heart Association Tobacco Regulation and Addiction Center, Dallas, TX USA; 3 Department of Medicine, MedStar Union Memorial Hospital, Baltimore, MD USA; 4 University of Louisville School of Medicine, Louisville, KY USA; 5 Department of Medicine, Vanderbilt University Medical Center, Nashville, TN USA

**Keywords:** E-cigarettes, Disposable, Flavors, Food and Drug Administration, Puff Bar, Google Trends

## Abstract

**Background:**

The impact of the U.S. Food and Drug Administration's (FDA) initial enforcement policy on flavored cartridge-based e-cigarettes and subsequent notice for the removal of flavored disposable electronic cigarettes (e-cigarettes) such as Puff Bar from the market has not been well evaluated. We, therefore, sought to examine the impact of the e-cigarette flavor-related policy changes on the online popularity of Puff Bar, a prototypic disposable e-cigarette.

**Methods:**

We tabulated the total weekly Google search queries originating from the U.S. for "Puff Bar" and "Puff Bars" from January 1, 2019, to December 31, 2021. We divided the three years into four (4) distinct periods using the dates of the initial announcement to ban non-tobacco flavored e-cigarettes (September 11, 2019), the finalization of the FDA enforcement policy on cartridge-based flavors (January 2, 2020), and the notice for the market withdrawal of flavored disposable e-cigarettes (July 20, 2020) as reference time points. Then, we used piecewise linear regression and autoregressive integrated moving average (ARIMA) to compare the trends in searches for Puff Bar for the four (4) periods.

**Results:**

Before the initial announcement to ban non-tobacco flavored e-cigarettes, online search queries (per 10 million Google searches) for Puff Bar were slowly rising at a rate of 0.58 queries per week (95%CI: -0.80 – 1.97). Following the announcement, searches for Puff Bar increased significantly at a rate of 16.61 queries per week (95%CI: 12.13 – 21.10). The rate of searches for Puff Bar then increased exponentially at 40.08 queries per week (95%CI: 27.32 – 52.84) following the FDA flavor ban, which excluded disposable e-cigarettes. Then, the rate of increase declined but remained relatively stable at 3.67 queries per week (95%CI: 0.69–6.65) until the FDA's notice to remove flavored Puff Bar products from the market. Following this notice, the rate of searches for Puff Bar significantly declined (rate: -4.97 queries per week; 95%CI: -5.40—-4.54).

**Conclusions:**

The tracking of online search data demonstrates rapid public recognition of the FDA's announcements of tobacco regulatory actions.

## Background

Electronic cigarettes (e-cigarettes) are the most widely used tobacco products among adolescents and young adults [1]. Despite the recent decline in e-cigarette use, in 2021, an estimated 320,000 U.S. middle school students and 1.72 million high school students reported using e-cigarettes in the past 30 days [1]. Most adolescents and young adults cite flavors as their reason for e-cigarette use and report fruit, candy, and desserts as their most commonly used flavors [1, 2].

As part of its efforts to curb the youth e-cigarette epidemic, the U.S. Food and Drug Administration (FDA) finalized an enforcement policy on flavored cartridge-based e-cigarettes in January 2020 [3], following the initial announcement to ban non-tobacco flavored e-cigarettes in September 2019 [4]. The initial FDA flavor ban excluded disposable flavored e-cigarettes such as Puff Bar, which was sold in multiple youth-friendly flavors at the time. Subsequently, in July 2020, the FDA notified companies, including the manufacturers of Puff Bar and other disposable e-cigarettes, to remove their flavored disposable products from the market [5]. After temporarily staying off the market, Puff Bar reemerged using synthetic nicotine formulation to circumvent FDA regulation. Although a prior study indicated that the initial flavor ban could prompt most e-cigarette users to replace their cartridge-based devices with disposable products [6], studies examining the direct impact of these policy changes on disposable e-cigarettes are limited.

Using traditional epidemiologic data to study rapidly evolving changes in population behavior and to evaluate the direct impact of policy changes can be challenging. However, online search data such as Google Trends provide real-time information on online search behaviors and have been used to monitor the public's interest in e-cigarettes and other consumer behaviors [7–9]. Such data can be used to examine changes in online search behaviors, a proxy of population interest and likely behavior [10, 11] immediately following health campaigns and policy implementation [12, 13]. In our current study, we evaluated the impact of the e-cigarette flavor-related announcements and policy changes on disposable e-cigarettes by examining trends in the Google search queries for Puff Bar among people in the U.S. who use Google search, the leading search engine used by the U.S. population [14].

## Methods

We examined the impact of the e-cigarette flavor-related policies on the online popularity of disposable e-cigarettes by examining the Google Search Query Data for Puff Bar, a prototypic disposable e-cigarette. We extracted the actual weekly Google search volume data for Puff Bar using the terms "Puff Bar" and "Puff Bars" from Google as query fractions per 10 million search queries. Weekly search volume data originating from the U.S. between January 1, 2019, to December 31, 2021, were retrieved and analyzed. We divided this period into four (4) distinct periods separated by three (3) key events: the initial announcement to ban non-tobacco flavored e-cigarettes (September 11, 2019), the FDA flavor ban which excluded disposable e-cigarettes (January 2, 2020) and the subsequent notification of the manufacturers of Puff Bar to remove their flavored products from the market (July 20, 2020). Period 1: January 1, 2019, to September 7, 2019; Period 2: The week of September 11, 2019, to December 28, 2019; Period 3: The week of January 2, 2020, to July 18, 2020; and Period 4: The week of July 20, 2020, to December 31, 2021.

### Statistical analysis

First, we plotted the weekly Google search volumes for Puff Bar over time to visualize the temporal trends in searches. Then, using piecewise linear regression with Puff Bar search queries (per 10 million google search queries) as the dependent variable and time (in weeks) as an independent variable, we estimated and compared the rate of Puff Bar searches for the four distinct periods. Also, utilizing data from Period 1 and using autoregressive integrated moving average (ARIMA), we forecasted the expected Google search volumes for Puff Bar for Periods 2, 3, and 4 had these events and policy changes not occurred.

The piecewise linear regression analyses were conducted using Stata version 16 (StataCorp, College Station, TX), and the ARIMA analyses were done with RStudio Version 1.4.1717. A 2-sided alpha (α) level of < 0.05 was used to determine statistical significance of the results.

## Results

**Fig. 1:**
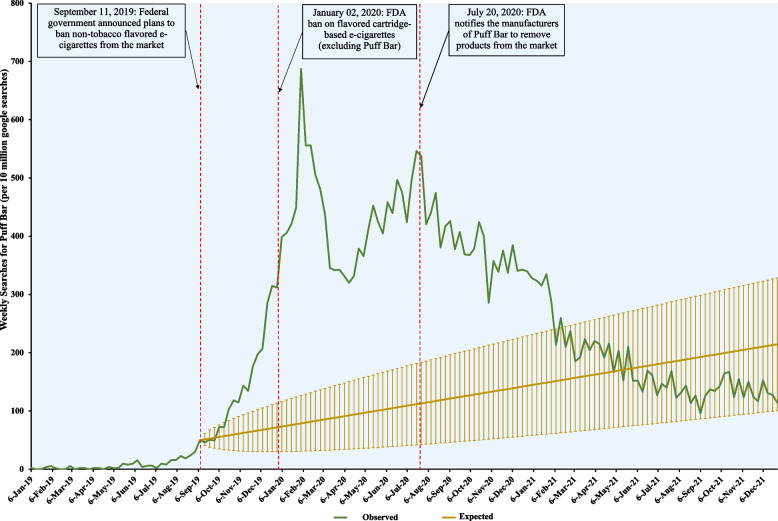
Trends in Google Searches for Puff Bar

Figure 1 shows the temporal trends in Google queries for Puff Bar between January 1, 2019, to December 31, 2021. Before the initial announcement to ban non-tobacco flavored e-cigarettes, online search queries (per 10 million Google searches) for Puff Bar were slowly rising at a rate of 0.58 queries per week (95%CI: -0.80 – 1.97). Following the announcement, searches for Puff Bar increased significantly at a rate of 16.61 queries per week (95%CI: 12.13 – 21.10). The rate of searches for Puff Bar then increased enormously at 40.08 queries per week (95%CI: 27.32 – 52.84) following the FDA flavor ban, which excluded disposable e-cigarettes. Then, the rate of increase declined but remained relatively stable at an average rate of 3.67 queries per week (95%CI: 0.69–6.65) – an initial decrease of -37.74 queries per week (95%CI: -55.51—-19.97) and a later increase of 11.56 queries per week (95%CI: 7.11–16.00)—until the FDA's notice to remove Puff Bar flavored products from the market. Following this announcement, the rate of searches for Puff Bar significantly declined (rate: -4.97 queries per week; 95%CI: -5.40—-4.54). These observed changes differed from the counterfactual scenario – i.e., the expected rate of Puff Bar searches had these events not happened (Fig. 1).

## Discussion

Using Google search query data, we found that online search popularity for Puff Bar increased significantly following the announcement to ban non-tobacco flavored e-cigarettes and the initial FDA flavor ban. However, Puff Bar-related searches declined substantially after the FDA notified manufacturers to remove the flavored products from the market.

The availability of youth-appealing flavored e-cigarettes is among the most cited reason for e-cigarette use among youth [2, 15, 16]. Most youth and young adults report their first e-cigarettes to be non-tobacco flavored [17]. The availability of flavors may therefore be fueling the youth e-cigarette epidemic. In addition, chemicals such as diacetyl and acetyl propionyl, toxic when inhaled, have been found in flavored e-liquids, raising serious concerns about their safety when used in e-cigarettes [18]. Therefore, the FDA's enforcement policy against flavored e-cigarettes has been perceived as a positive step toward combating the youth e-cigarette epidemic and protecting population health.

Few studies have examined the implications of the FDA's enforcement policies against flavored e-cigarette products. We found that Puff Bar-related searches increased significantly following the announcement on September 11, 2019, to ban non-tobacco flavored e-cigarettes. This may have been due to last-minute panic purchases and stockpiling of flavored e-cigarettes by some e-cigarette users prior to the finalization of the federal flavor ban [19]. Similar to findings from our study, a prior study that utilized Google relative search volume data also found that following the initial FDA flavor ban on January 2, 2020, the online popularity of JUUL, a prototypic cartridge-based e-cigarette, declined significantly [6]. In contrast, online searches for Puff Bar, a disposable e-cigarette, increased significantly over the same period [6]. This was attributed to a loophole in the initial policy, which excluded flavored disposable e-cigarettes [6]. Based on these findings, the authors concluded e-cigarette users might switch from cartridge-based e-cigarettes to disposable e-cigarettes to circumvent the initial flavor ban. Subsequent national surveys confirmed these findings as the use of disposable e-cigarettes among youth increased significantly, becoming the most common device used by youth [1, 20, 21].

While the study mentioned above used the publicly available Google Data, which are reported as scaled relative search volumes (0–100%) [6], we used the actual Google search query data in this study, which allow for more inferential analyses. We also extend the findings of the prior study to show that the online popularity of Puff Bar has significantly declined following the notice by the FDA to the manufacturers to remove their flavored products from the market [5]. Implications of the recent decline in online popularity for Puff Bar are unclear but may contribute to the overall decrease in e-cigarette use among youth as these flavored disposable products become less available.

Using Google data, we assessed the immediate impact of the FDA regulatory policies on online search behaviors of the U.S. population. We have shown the rapid public recognition of the FDA's announcements of tobacco regulatory actions and how these policies may influence online search behavior. This adds to the utility of online search query data to complement traditional epidemiologic survey data in monitoring changes in population behavior [22, 23]. Continued surveillance of online search data may help tobacco regulatory authorities to evaluate the immediate impact of tobacco-related policy changes and identify loopholes that may thwart their efforts to reduce tobacco use among youth.

The findings of our study should be interpreted cautiously, considering some limitations. First, although several brands of disposable cigarettes are on the market, we only examined one brand of disposable e-cigarettes, Puff Bar. Future studies are needed to assess the online popularity of other disposable e-cigarettes, especially now that the online popularity of Puff Bar has declined. Secondly, Google data does not allow subgroup analyses by important sociodemographic characteristics, including age. Hence, the impact of these policy changes, specifically on youth e-cigarette use, cannot be assessed. Also, while Google search is the leading search engine used by most U.S. population, about 13% of the population uses other search engines, hence not captured in our present study. Finally, we assessed the implications of the e-cigarette flavor-related announcements and policy changes only on the online popularity of Puff Bar and not on actual sales of these products. Studies are needed to assess how these policy changes have influenced sales, particularly black market sales of cartridge-based and disposable e-cigarettes.

## Conclusions

In conclusion, online search popularity for Puff Bar surged following the announcement to ban non-tobacco flavored e-cigarettes and the initial flavor ban, which excluded flavored disposable e-cigarettes. However, there has been a significant decline in Puff Bar-related searches since the FDA notified its manufacturers to remove the product from the market. Also, we show the rapid public recognition of the FDA's announcements of tobacco regulatory actions, adding to the utility of Google data for monitoring rapid changes in population behavior and hence in tobacco regulatory science.

## Data Availability

The publicly available data can be found here (https://trends.google.com/trends/?geo=US). However, the search volumes used in the study were obtained directly from Google through a special agreement and will be made available upon reasonable request.

## Abbreviations

ARIMA: Autoregressive Integrated Moving Average
FDA: Food and Drug Administration
U.S.: United States
